# Ultra‐low background Raman sensing using a negative‐curvature fibre and no distal optics

**DOI:** 10.1002/jbio.201800239

**Published:** 2018-11-19

**Authors:** Stephanos Yerolatsitis, Fei Yu, Sarah McAughtrie, Michael G. Tanner, Holly Fleming, James M. Stone, Colin J. Campbell, Tim A. Birks, Jonathan C. Knight

**Affiliations:** ^1^ Department of Physics University of Bath Bath UK; ^2^ School of Chemistry University of Edinburgh Edinburgh UK; ^3^ EPSRC IRC Hub, Centre for Inflammation Research, Queen's Medical Research Centre University of Edinburgh Edinburgh UK; ^4^ Scottish Universities Physics Alliance (SUPA), Inst. of Photonics and Quantum Sciences (IPaQS) Heriot‐Watt University Edinburgh UK

**Keywords:** fibre fabrication, hollow‐core fibre, Raman fibre probe, Raman sensing

## Abstract

Measuring Raman spectra through an optical fibre is usually complicated by the high intrinsic Raman scatter of the fibre material. Common solutions such as the use of multiple fibres and distal optics are complex and bulky. We demonstrate the use of single novel hollow‐core negative‐curvature fibres (NCFs) for Raman and surface‐enhanced Raman spectroscopy (SERS) sensing using no distal optics. The background Raman emission from the silica in the NCF was at least 1000× smaller than in a conventional solid fibre, while maintaining the same collection efficiency. We transmitted pump light from a 785‐nm laser through the NCF, and we collected back the weak Raman spectra of different distal samples, demonstrating the fibre probe can be used for measurements of weak Raman and SERS signals that would otherwise overlap spectrally with the silica background. The lack of distal optics and consequent small probe diameter (<0.25 mm) enable applications that were not previously possible.

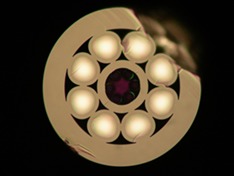

## Graphical abstract

Measuring Raman spectra through an optical fibre is usually complicated by the high intrinsic Raman scatter of the fibre material. Common solutions such as the use of multiple fibres and distal optics are complex and bulky. A single hollow‐core negative‐curvature fibre is used for Raman and surface‐enhanced Raman spectroscopy sensing with no distal optics. This new optical fibre shows a 1000× background reduction compared with conventional fibres.

## 1 INTRODUCTION

Fibre‐based Raman spectrometers are widely available and are used in endoscopy. However, when light from a spectrally narrow pump passes through an optical fibre, an undesirable “background” Raman Stokes‐wave signal is generated in the fibre by the silica glass from which it is made. This background can be large enough to overwhelm any signal collected from a biological sample of interest at the distal end of the system. For example, many useful carbon‐based Raman bands overlap spectrally with this background. For this reason, such systems use different fibres for excitation and collection in order to spatially separate the background from the collected signal. This is problematic because the two fibres do not physically point at the same position and distal optics are required to correct for this 1, as well as to filter out the fibre‐generated Raman signal. This increases the size of the probe. When working in microscopic systems, for example, in the distal lung, inside the brain or in blood vessels, additional optics and filters cannot be accommodated because they are too large.

The silica background can be reduced by using hollow‐core fibres (HCF). The mode of an HCF is guided in air and interacts very little with the silica, greatly reducing the background 2. Previous schemes used HCFs with complex structures such as kagome 3 or photonic‐bandgap fibres 4. They suffered from reduced collection efficiency, either due to the use of a spatial filter to suppress the background 3 or a large separation of the HCF and the collection fibres 4. In 3, to solve this, distal optics were used to couple light efficiently back into the fibre.

Similar HCFs, but incorporating a microstructured outer cladding to collect the light from the distal end, have been reported for Coherent Anti‐Stokes Raman Spectroscopy (CARS) experiments 5, 6. Guidance in air rather than glass again reduces degradation of the pump light (though the degradation mechanisms for CARS are different), so we expect that such fibres could suppress the silica background for ordinary (ie, Stokes‐wave) Raman spectroscopy as well. However, the continued presence of an extensive kagome or photonic‐bandgap structure in the inner cladding not only limits collection efficiency (by distancing the collection waveguide from the central excitation core), but also makes fibre fabrication quite challenging as the different air‐silica guiding structures need to be controlled simultaneously during the draw.

Allowing a fluid sample to fill a hollow core by capillary action has been reported to increase the Raman signal 7, but such a scheme is unsuitable for endoscopy and depends on the refractive properties of the fluid.

**Figure 1:**
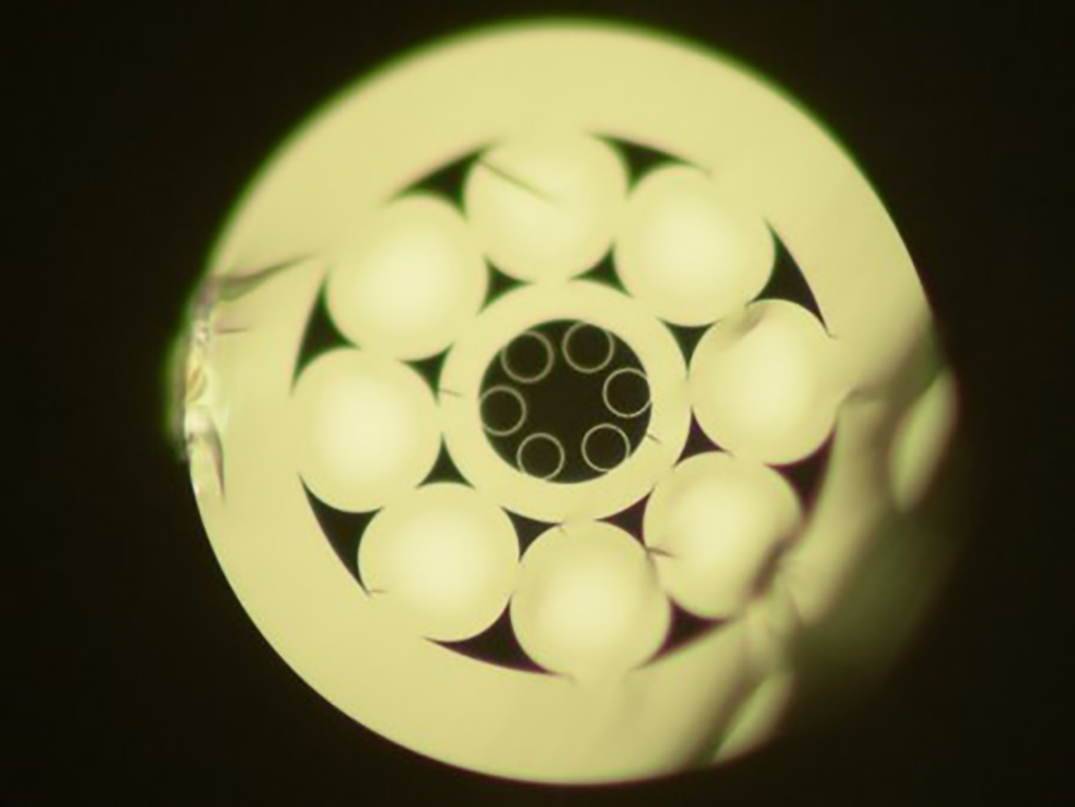
Optical micrograph of the NCF, illuminated by reflected light. The hollow core is bounded by a ring of six capillaries. Surrounding these is a ring of eight solid multimode cores. The outer diameter of the fibre is 210 μm and the core diameter is 20 μm

Here, we report the use of a hollow‐core negative‐curvature fibre (NCF) for delivering pump light for Raman spectroscopy. The guiding structure is much simpler than a kagome or photonic‐bandgap fibre, with just a single ring of six nontouching capillary resonators surrounding an empty core. These can be seen in the middle of Figure 1. As understood from the anti‐resonant reflecting optical waveguide (ARROW) model 2, 8, the walls of the capillaries act as Fabry‐Perot resonators, expelling light (and thus confining it to the core) at their anti‐resonant wavelengths. The thickness of the walls determines the low‐loss passbands of the fibre. The negative curvature of the core boundary (convex as seen from the centre, unlike the concave boundary of an ordinary circular core), and the separation of the capillaries from each other, together reduce the overlap of the guided mode with the silica glass to values as low as 10^−4^ in theory 2. In practice, the measured silica background was an order of magnitude lower than the best previously reported in HCF 3, and over 1000× lower than from a solid‐core fibre.

The fibre also incorporates an outer ring of eight large conventionally guiding multimode cores to collect the Raman signal. The simple inner cladding of the NCF region allowed the collection cores to be located closer to the central excitation core than is possible in kagome or photonic‐bandgap fibres 5, 6, increasing the collection efficiency, while still allowing enough separation to keep stray pump light out of the collection cores. Thus, we achieved a similar collection efficiency to that obtained by pumping via a solid fibre, but without the use of distal optics 3, 9 or other fibres 4, 10, 11.

As a result, using a single fibre with multiple cores, we were able to measure the weak Raman signals that overlap spectrally with the silica background. In doing so, we reduced the transverse probe dimension from a millimetre or more in previous manifestations to around 210 μm.

## 2 HOLLOW‐CORE NCF

We fabricated the fibre by the stack‐and‐draw technique 12 (see Figure 1). The inner region consists of a single ring of six silica capillaries around the hollow core. The hollow core is designed to guide light of wavelength 785 nm and is 20 μm in diameter. Surrounding this region is an additional ring of eight multimode graded‐index Ge‐doped cores, designed to collect the Raman signal from a distal sample. The peak numerical aperture (NA) of these cores is 0.3 and their diameter is 28 μm. The outer diameter of the fibre is 210 μm.

**Figure 2:**
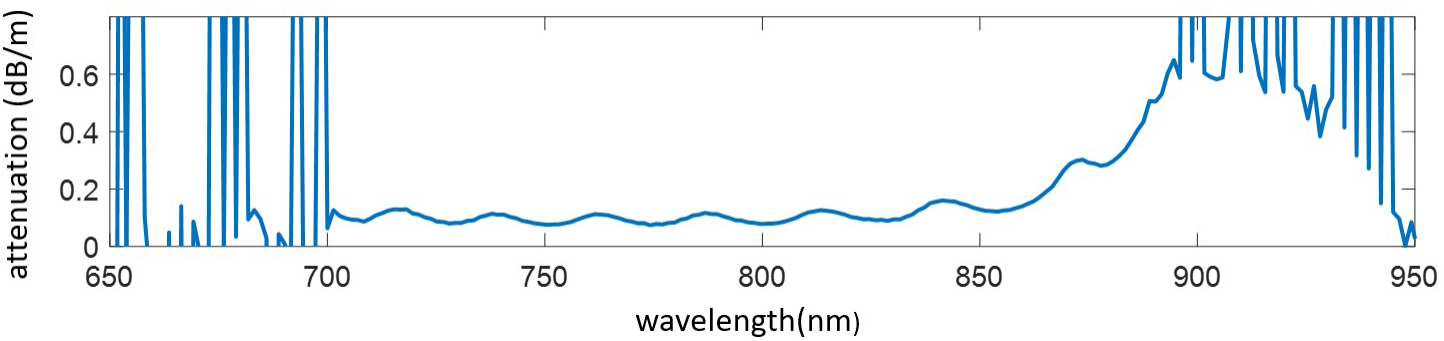
Spectral attenuation of the fibre's hollow core

The cutback method was used to determine the transmission band and attenuation of the fibre. White light was launched into the hollow core of 60 m of the fibre, and the transmission was recorded using an optical spectrum analyser. The fibre was cut back to 10 m and the transmission was recorded again. Figure 2 shows the resulting attenuation spectrum of the hollow core. The fibre transmits with low loss, and hence low interaction of the light with the glass, from well below 785 nm. The attenuation is 0.1 dB/m at the pump wavelength of 785 nm.

## 3 EXPERIMENTAL SETUP—SURFACE‐ENHANCED RAMAN SPECTROSCOPY SAMPLE

**Figure 3:**
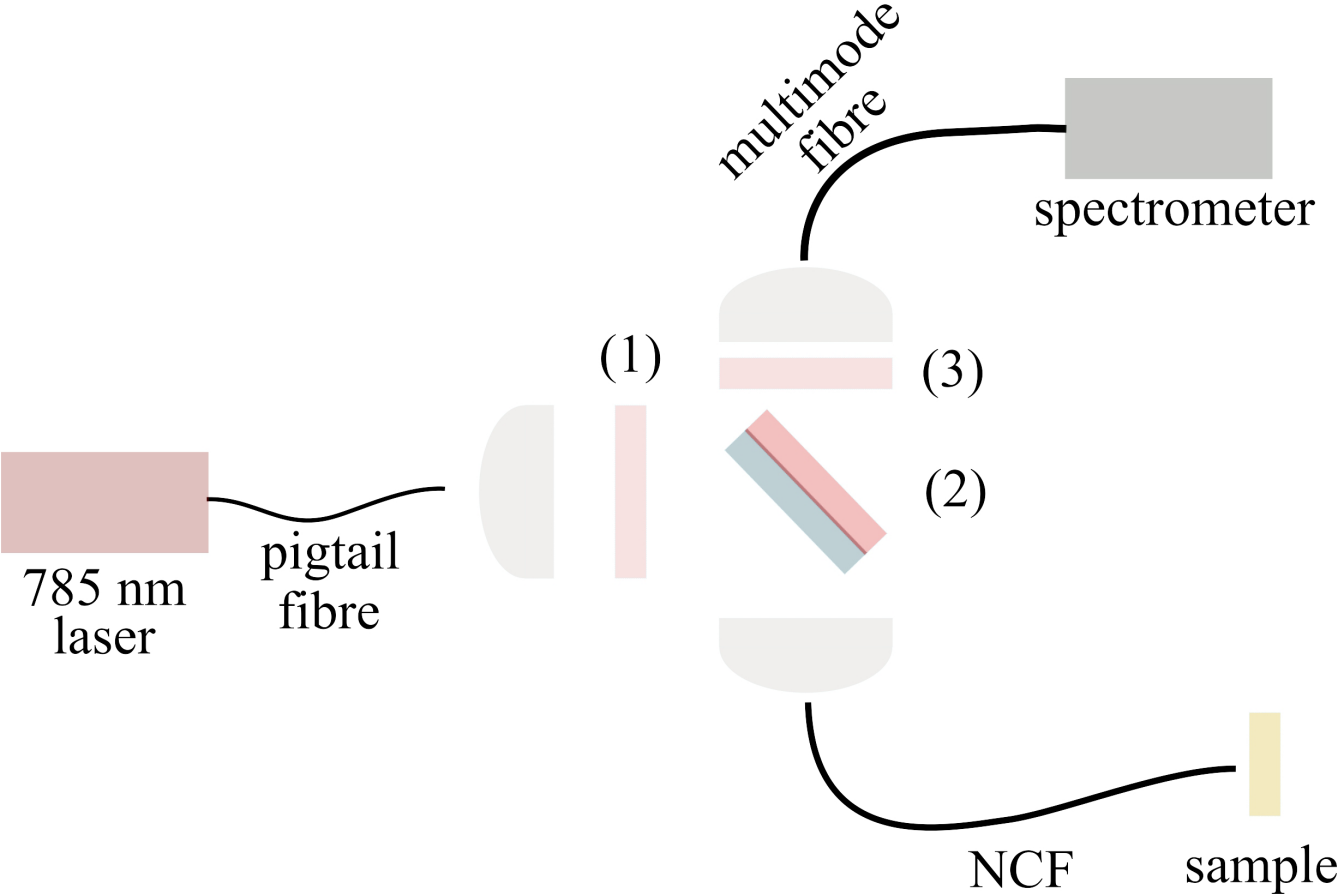
Experimental setup where (1) is a 785‐nm bandpass filter (2) is a 785‐nm dichroic mirror and (3) is a 791.6‐nm longpass filter

Tests were carried out with approximately 1 meter of the fibre, coiled to a radius of around 15 cm. The experimental setup is shown in Figure 3. Light from a single‐mode fibre pigtailed 785‐nm diode laser was coupled to the proximal end of the NCF via a bandpass filter (to remove any silica background generated in the pigtail fibre) and a dichroic mirror (to separate any Raman signal returned by the fibre from the pump signal). A longpass filter in the collection arm filters out any residual pump. A multimode fibre with a graded‐index core of 100‐μm diameter (peak NA = 0.3) was used to collect the returned light from the entire proximal endface of the NCF (ie, both the hollow core and all of the Ge‐doped solid cores) and deliver it to the fibre‐coupled spectrometer (Avantes SensLine Hero, Apeldoorn, Netherlands). After careful alignment, the coupling efficiency from the 785‐nm laser into the hollow core was more than 90%. The laser power was chosen so that 1.2 mW of pump light was transmitted to the distal end.

The NA of the HCF is consistent with an estimate of 0.05 assuming diffraction from a circular aperture, so we used a low‐NA (0.15) lens to couple the laser light. The higher NA of the collection cores means that some of the back‐scattered Raman light delivered by them is not captured by the lens. However, this light would be lost anyway due to the 0.13 NA of the spectrometer. The integration time of the spectrometer was set to 1 s.

**Figure 4:**
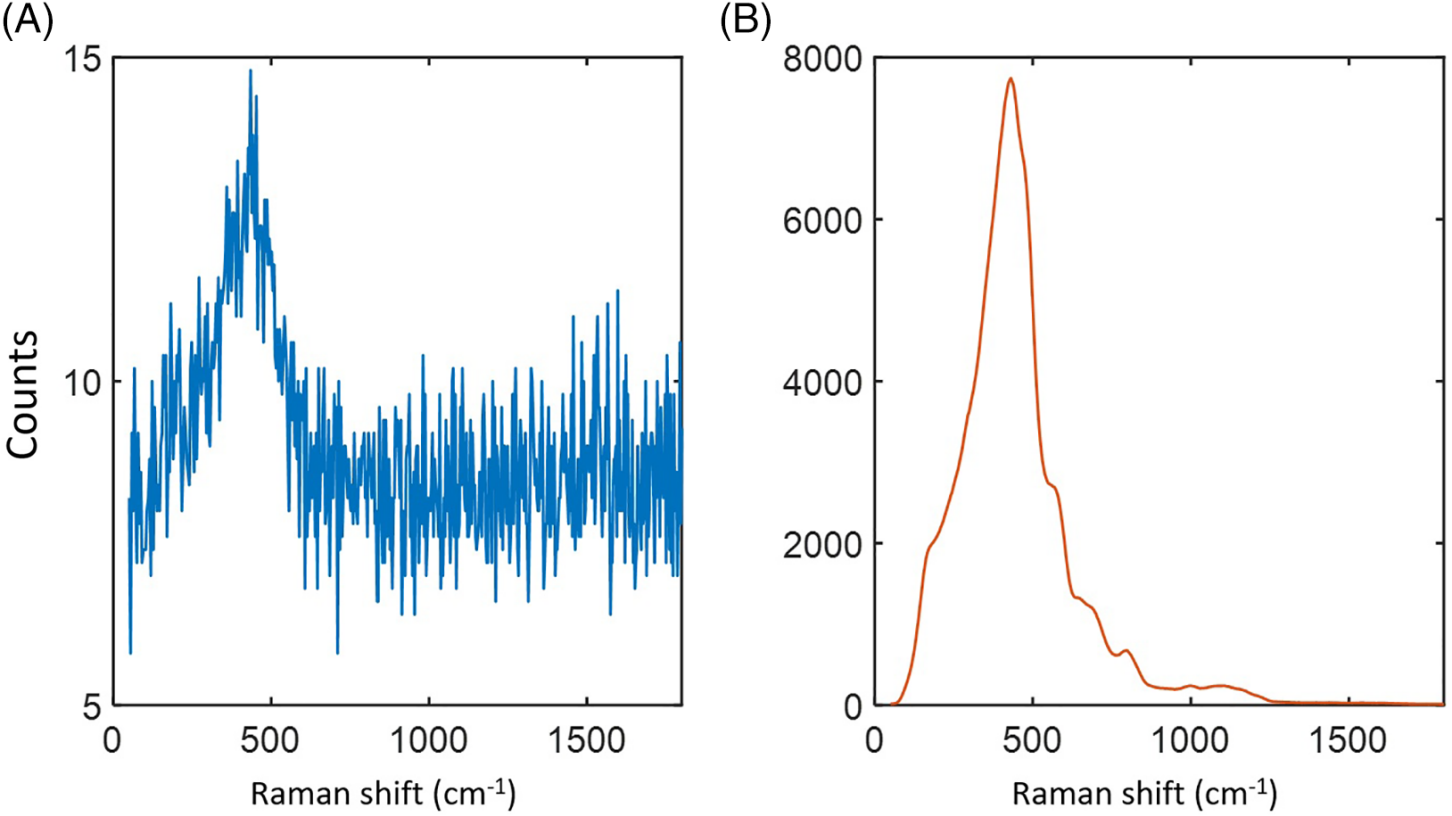
Generated silica background when pump light was coupled to (A) the hollow core (blue) and (B) the solid core (orange). Note the different vertical scales (1‐s integration time)

To demonstrate the reduction of silica background, the Raman response was first measured without any sample at the distal end of the fibre. To compare this with the performance of a solid‐core fibre, we simply coupled the pump light into one of the Ge‐doped collection cores instead of the hollow core and repeated the measurement. Figure 4 shows the Raman responses of the hollow core and the solid core. There is a 1000× reduction of the background when the pump light was coupled to the hollow core compared to the solid core. We believe that the remaining background is due to an imperfect match between the modes of the NCF and the laser's pigtail fibre, causing stray light that is coupled into the glass rather than the hollow core. Perfecting this match is necessary to realise the 10^4^× reduction given in theory by the overlap of a pure NCF mode with the glass 2.

**Figure 5:**
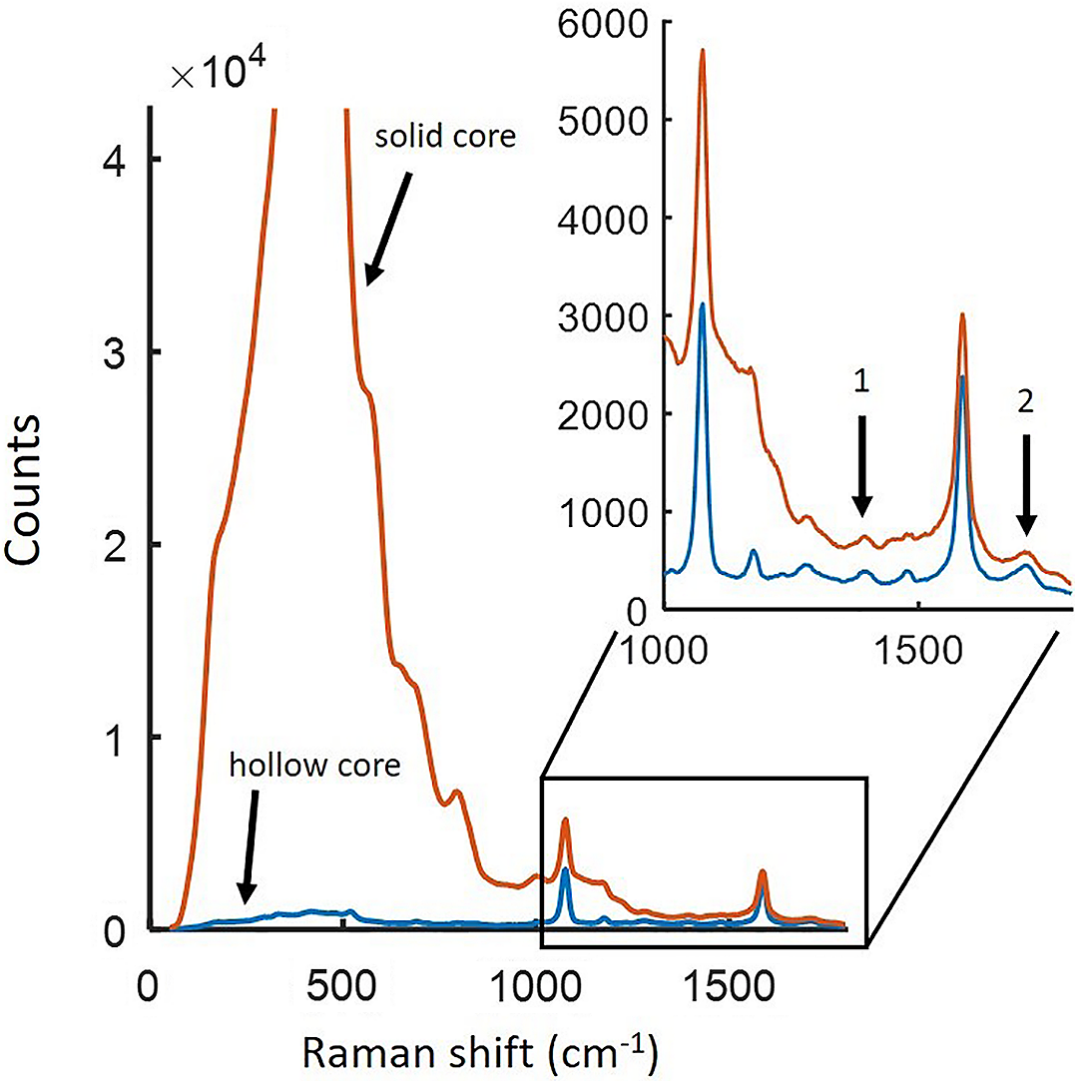
The SERS signal from the sample when pumped via the hollow core (blue) and the solid core (orange), 1 and 2 are the MBA SERS bands used for pH sensing (10‐s integration time)

We then placed a (dry) surface‐enhanced Raman spectroscopy (SERS) sample at the distal end of the NCF. The sample comprised paramercaptobenzoic acid (pMBA) labelled gold nanoparticles deposited on a gold‐coated glass chip on a glass microscope slide. SERS spectra were recorded when pumped via the hollow core and one of the solid collection cores in turn. Figure 5 shows the collected signals, which include peaks that match the published spectrum of mercaptobenzoic acid (MBA) 13, 14. The pMBA peaks around 1050 and 1550/cm have similar heights above the background in both cases, indicating that the collection efficiency is similar when pumped via the solid core and the hollow core. However, the background is much reduced when pumped via the hollow core. MBA can be used for pH sensing 13, 14 by measurement of the ratio between the intensity of the two small peaks (labelled 1 and 2) around 1380 and 1700/cm. Eliminating the silica background through use of the NCF should improve the accuracy of this measurement 14.

## 4 INTRINSIC RAMAN

In the previous section, we used an SERS sample to characterise the fibre. The SERS sample is engineered in such way to have a strong Raman response. Typical SERS samples have 10^6^ stronger response compared to any intrinsic Raman sample 15. It is far more difficult to observe an intrinsic Raman response. To strengthen the detected spectrum (and increase total counts), we therefore increased the laser power from 1.2 to 6 mW and set the spectrometer integration time to 10 s. Nevertheless, these are still low powers and integration times compared to the literature.

**Figure 6:**
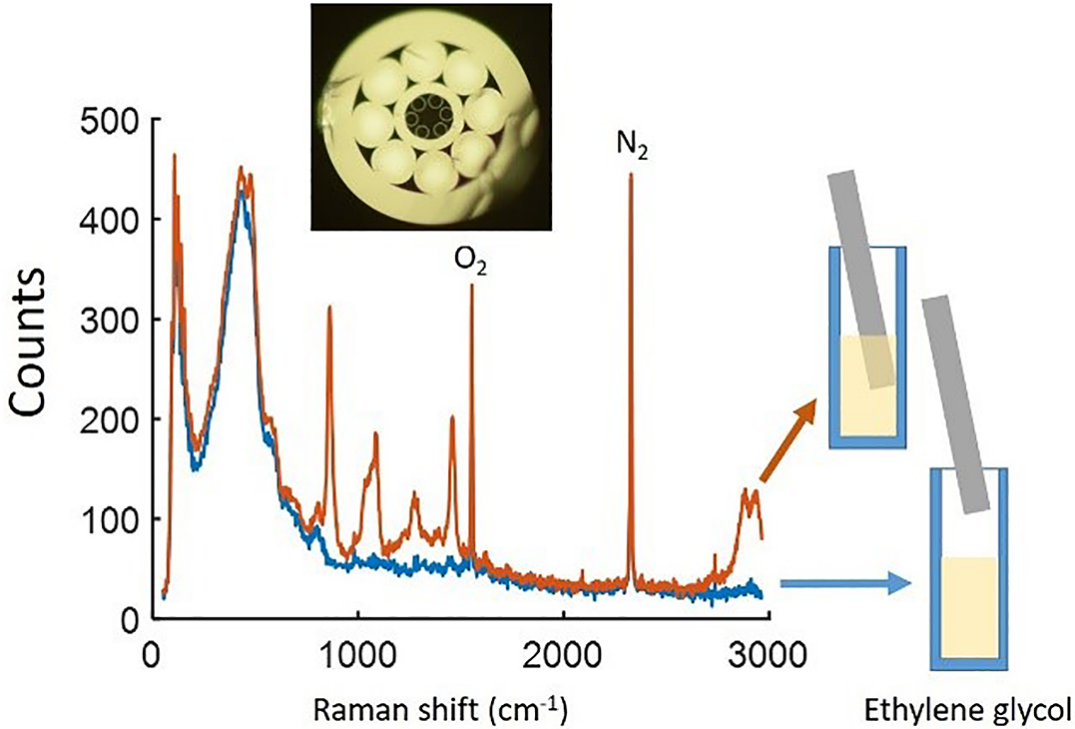
The collected signal when pump light is coupled to the hollow core before (blue) and after (orange) the fibre tip was immersed in ethylene glycol (10‐s integration time)

A solution of ethylene glycol was used to test the capability of the fibre. A thin film of household plastic wrap (10‐15 μm thick) was used to seal the end of the fibre and prevent the hollow core filling with liquid. (Measurements of the background spectrum before and after fitting the film were indistinguishable.) The response when pumped via the hollow core is shown in Figure 6 both before (blue) and after (orange) the fibre was immersed into the solution. We observed clearly strong peaks at 865.6, 1089.5, 1272 and 1463/cm that relate to ethylene glycol 16. (The peaks, visible in both measurements, at 1555 and 2331/cm, are the molecular oxygen and nitrogen peaks, respectively, from the atmospheric air in the hollow core of the fibre 17. These peaks were not visible in Figure 5 as the collected SERS signal is stronger compared to any intrinsic Raman but they are visible in all the following figures.)

**Figure 7:**
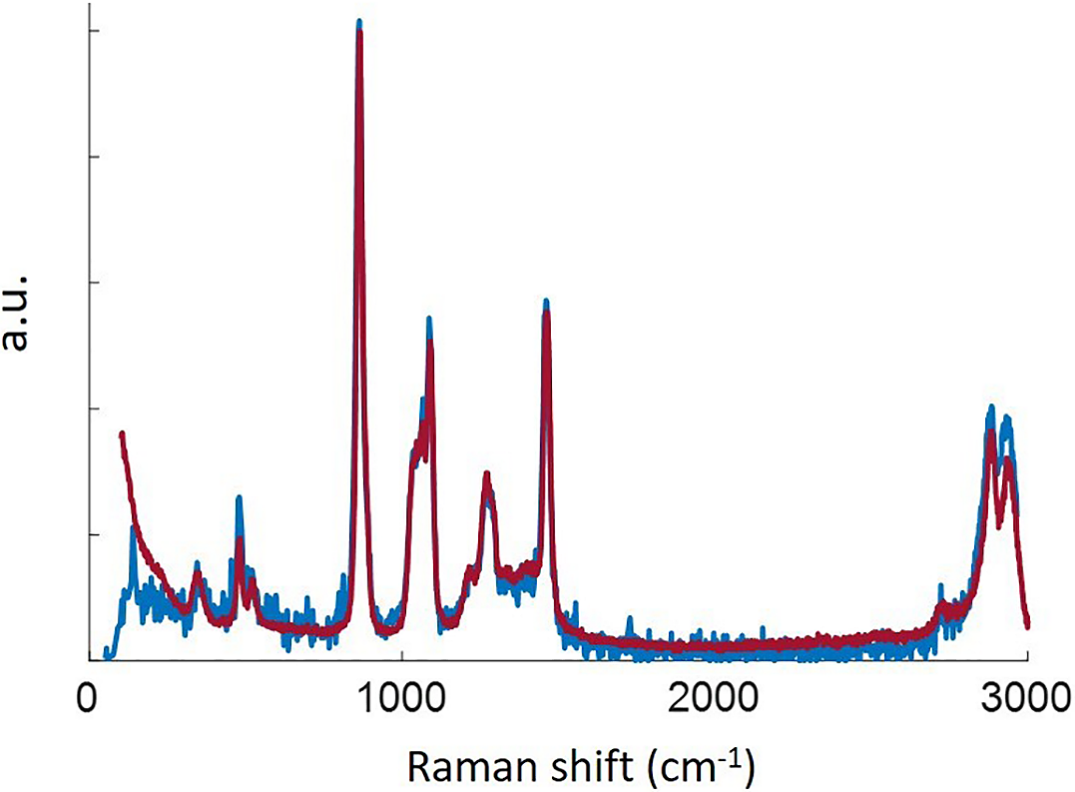
(Blue) Signal from ethylene glycol obtained by subtracting the two plots in Figure 6. (Purple) Signal from ethylene glycol collected using a commercially available Raman microscope. The vertical scale is linear but in arbitrary units because we are comparing two different spectrometers; the spectra are scaled so that the peak values match

Subtracting the remaining background from the collected signal (the blue and orange curves in Figure 6), we can remove the remaining features caused by the fibre, Figure 7(blue), and can now also observe the weaker peaks of ethylene glycol at 481.4 and 523.6/cm. We verified our results using a Renishaw inVia confocal Raman microscope with a 785‐nm laser, Figure 7 (purple); the two plots match. This demonstrates the ability of the fibre probe to be used for intrinsic Raman sensing, collecting the response from samples that produce Raman signals close to the silica background.

**Figure 8:**
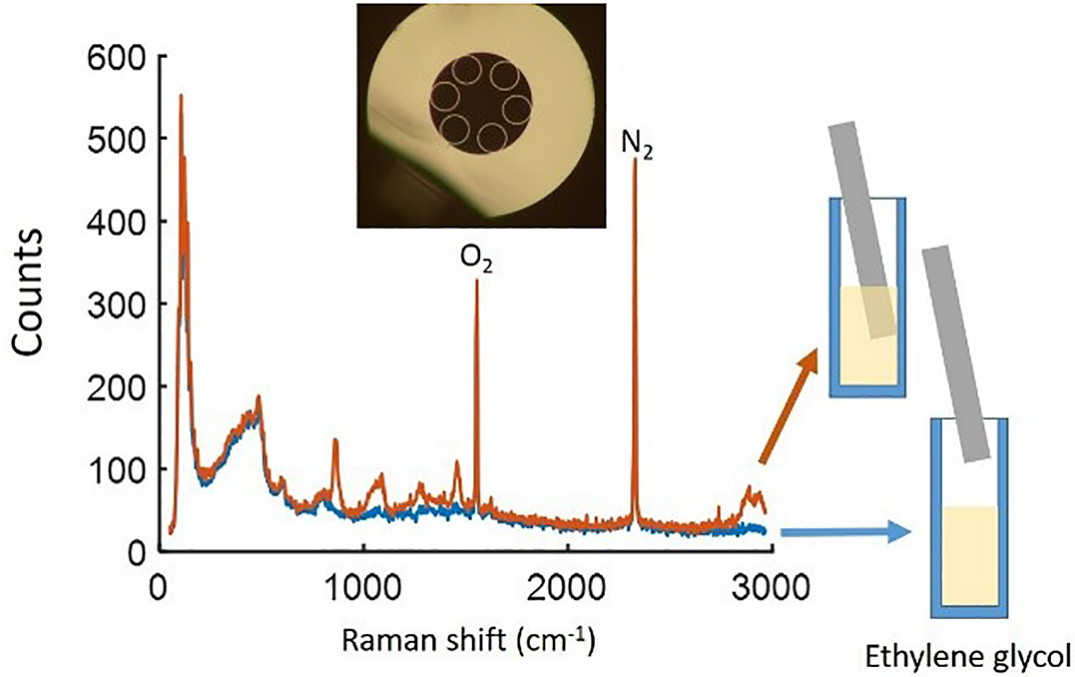
The collected signal when pump light is coupled to the hollow core before (blue) and after (orange) a fibre without Ge‐doped collection cores was immersed in ethylene glycol (10‐s integration time)

To test the increase in collection efficiency due to the use of the Ge‐doped ring of cores, a similar NCF was made with just the hollow core and without any Ge‐doped cores. The above procedure was followed, and the fibre was immersed in ethylene glycol. The response is shown in Figure 8. The oxygen and nitrogen peaks (at 1555 and 2331/cm) from air in the fibres remained the same, confirming that the hollow cores in the two fibres were comparable. However, the height of the ethylene glycol peak at 865.6/cm decreased by 2/3, indicating a 3× increase in collection efficiency when the collection cores were present. The collection efficiency depends on the overlap between the acceptance‐angle cones of the (low‐NA) hollow core and the collection cores. While it can be improved by bringing the cores closer together, this may increase the silica background if stray pump light at the proximal end is coupled into the collection cores.

## 5 TISSUE RAMAN

**Figure 9:**
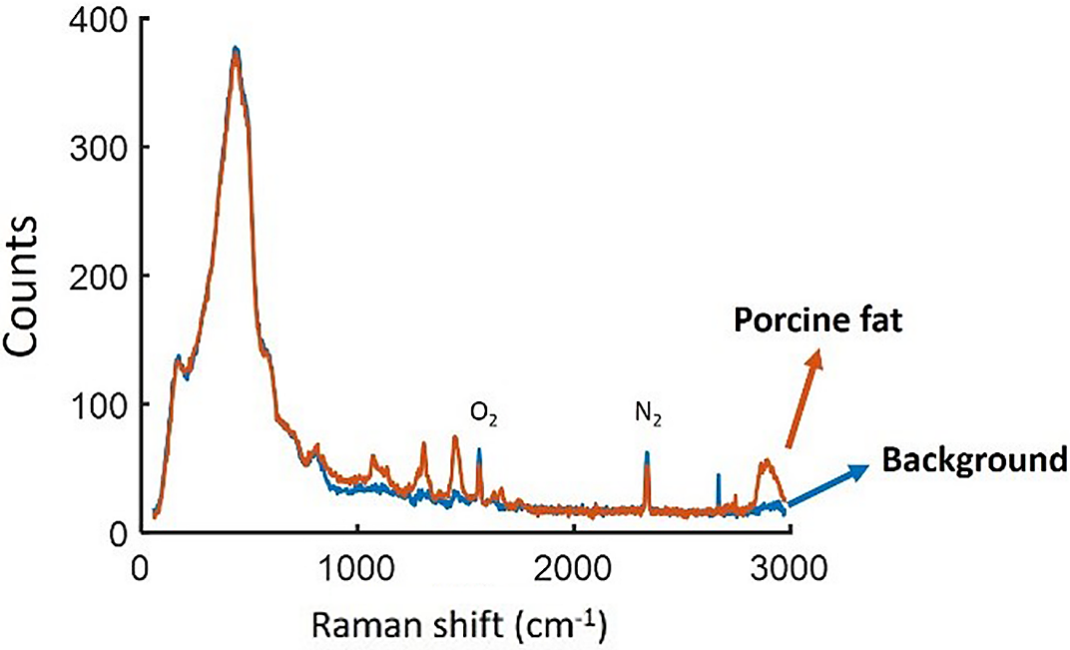
The collected signal when pump light is coupled to the hollow core before (blue) and after (orange) the fibre was in contact with the porcine fat (10‐s integration time)

Raman spectroscopy has been used in‐situ as a tool for medical diagnostic such as detecting pre‐cancerous tissue 10, 18. A portable *in vivo* tool that enables the on‐site detection of pre‐cancerous tissue will be advantageous for clinicians. Observing the Raman response from a tissue sample is difficult as the light penetrates only a few centimetres or even millimetres into the sample depending on the wavelength 19, giving a small volume of interaction. To demonstrate the ability of the fibre to be used for endoscopic Raman sensing, a piece of porcine tissue was used as a sample. The end of the fibre was again sealed using plastic wrap. The sample was placed on a microscope slide and the same procedure as for the SERS sample was followed. The Raman response from the sample is recorded and shown in Figure 9.

**Figure 10:**
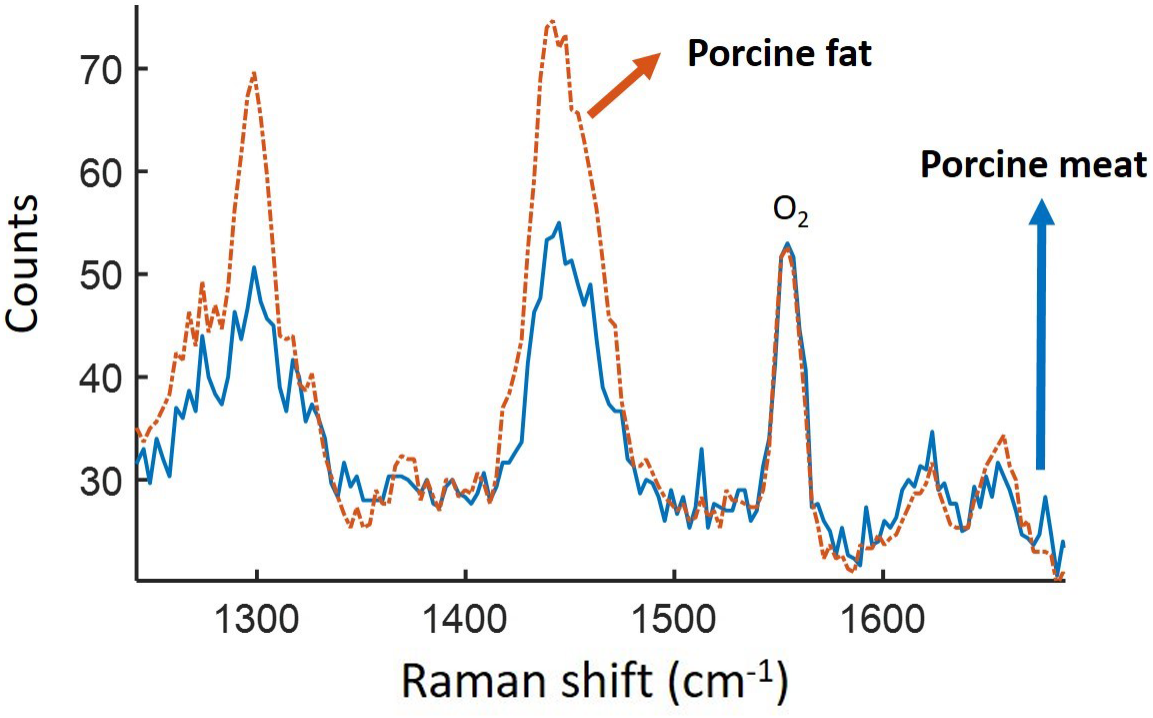
The collected signal when the fibre was in contact with porcine fat (orange) and porcine meat (blue) (10‐s integration time)

In Figure 9, there are clear peaks from the tissue sample (such as at 1063, 1128, 1274, 1299, 1442, 1658 and 1742/cm) which can be related to protein and lipid Raman bands 20 and match the published spectrum of porcine tissue 21. The ratio between the observed Raman peaks changes for different areas of the sample (eg, the two peaks at 1270 and 1300/cm), that is, for different lipid to protein ratios 21 as shown in Figure 10.

To our knowledge, this is the first demonstration of the detection of Raman signals from tissue samples obtained by a single fibre of such small size without distal optics and shows that the fibre probe can be used for endoscopic sensing. A previous scheme 10 incorporated multiple fibres and distal filtering, resulting in an overall probe diameter of 1.8 mm, compared to 0.2 mm here. The measurement was also enabled by the use of a 785‐nm laser: although the use of shorter wavelengths increases the Raman response 20, it increases the autofluorescence response of the tissue as well, overwhelming the Raman response 22.

**Figure 11:**
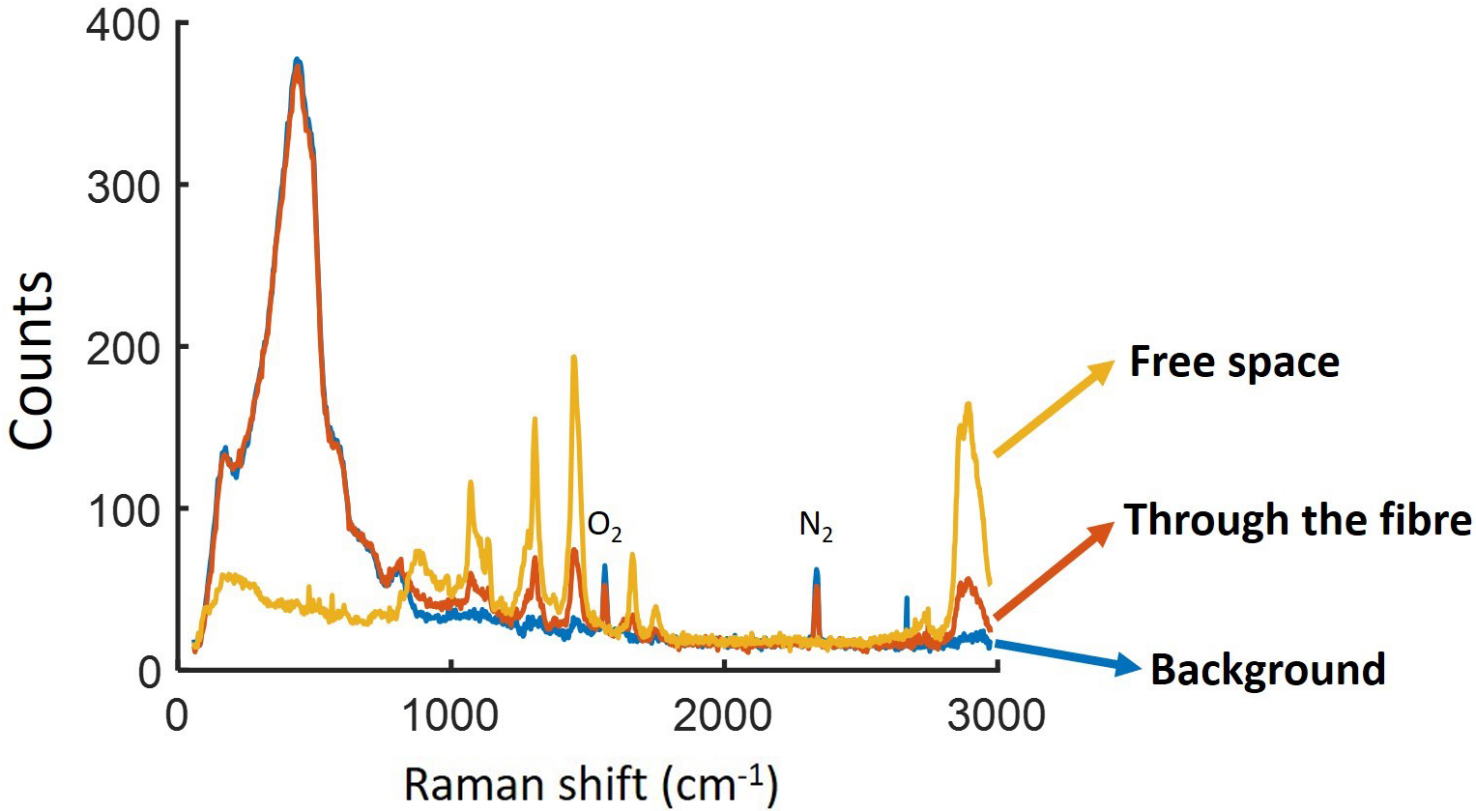
Generated silica background when pump light is coupled to the hollow core (blue). The collected signal through the fibre (orange) and in free space (yellow) from a porcine sample (10‐s integration time)

We tested the collection efficiency of the fibre by bypassing the NCF and placing the sample just after the lens used to couple light in to the NCF (Figure 3). The recorded response is shown in Figure 11. The fibre maintains a good collection efficiency of the Raman signal as only 2/3 of the light is lost through the fibre and free space. Without the collection cores, this loss would be much bigger.

For the intrinsic Raman experiments, we used short integration times (10 s) and low laser powers (6 mW through the fibre). With these settings, we can still resolve clear Raman features for both ethylene glycol and porcine tissue, demonstrating the efficiency of our system. Both of these parameters can be increased to give a larger signal.

## 6 CONCLUSION

We have demonstrated the use of negative‐curvature HCFs for Raman sensing by designing a portable highly efficient Raman probe. The generated silica background is an order of magnitude smaller compared to other schemes with HCFs 3, 4, while maintaining the same collection efficiency as a solid fibre. This was achieved without the use of any distal optics or additional fibres. We have demonstrated the use of this setup for measuring the SERS and intrinsic Raman response of different samples. A tissue sample was also used to demonstrate the ability of this system as an in vivo tool for endoscopic Raman sensing.

Data underlying the results presented in this paper are available from https://doi.org/10.15125/BATH-00559.
